# Calcium Phosphate Bone Cements Including Sugar Surfactants: Part One—Porosity, Setting Times and Compressive Strength

**DOI:** 10.3390/ma3104695

**Published:** 2010-09-30

**Authors:** Ariane Bercier, Stéphane Gonçalves, Olivier Lignon, Juliette Fitremann

**Affiliations:** 1 Université de Toulouse, Laboratoire des IMRCP, CNRS-Université Paul Sabatier, Bâtiment 2R1, 118 Route de Narbonne, 31062 Toulouse Cedex 9, France; 2 Teknimed SA, 11 rue Apollo, ZI Montredon, 31240 L’UNION, France

**Keywords:** calcium phosphate, surfactant, sugar, porosity, image analysis, setting time, cohesion

## Abstract

Addition of sugar surfactants, sucrose fatty acid esters and alkylpolyglucosides, to calcium phosphate cement designed for bone reconstruction is described. Thanks to their surface activity and through their adsorption at the surface of the calcium phosphate particles, they both induced a strong increase in the porosity (quantified by Image Analysis) and brought a very good workability. Other properties typically studied for these cements are reported, including setting times, compressive strength, cohesion in water, and effect of sterilization on these properties. The whole study brought good insight in the interest of adding these mild surfactants to improve several properties of the calcium phosphate cement, without impairing their function.

## 1. Introduction

Calcium phosphate bone cements are now well recognized materials for their usefulness in surgical practice for bone reconstruction [1,2,3,4,5,6]. Interest in these materials is due to the following properties. First, their mineral composition tends to be very close to the mineral composition of the bone itself: after setting and mineral transformation, cements transform in hydroxyapatite, which make them excellent biocompatible materials. Secondly, they can be prepared as a paste, so that surgeons can precisely fill bone defects, before hardening *in situ*. This setting is not achieved by heating as is the case with acrylic cements which are harmful for surrounding cells. Thirdly, calcium phosphate cements are osteoconducting materials, and tend to resorb to the benefit of true bone, but the quality and extent of cell colonization and bioresorption depends greatly on the porosity. However, the main drawback is their lack of mechanical resistance, making them only useful for bones not subjected to high loads.

While several formulations are offered on the market, some properties need to be improved and drawbacks need to be resolved [1,5]. Most especially, many studies focused on increasing the porosity of these cements. While they display a high microporosity, it is not enough to ensure a good bioresorption. Indeed, it has been shown that the nature of the cells which colonize and resorb the cement depends on the size of the pores. In order to get bone, inter-connected pores larger than 100 μm are more suitable. Different strategies have been set up to create pores in cements, including mainly the addition of water soluble and biocompatible crystals (sugars, mineral salts) and effervescent additives. The first strategy tends to impair injectability and setting while the second leads to risks associated with sudden gas release in body fluids. A third strategy, using surfactants as air‑entraining agents, which has been less used to date, is at the basis of this paper and will be presented in more detail below.

The second most desirable property for these cements made *in situ* is injectability. If the cement paste can be injected through a cannula, it is the least invasive surgical technique. As recently reviewed [6,7], inorganic additives or polymers can be added to improve injectability. Polymers especially are used to increase the viscosity of the aqueous phase, thus decreasing the filter pressing phenomenon. However, the adsorption phenomena of the organic additives on solid particles that could bring a lubricating effect, especially in this context of highly concentrated dispersions, are neglected [8].

When properties are tuned by changing mineral components or by adding different organic additives, there is always a compromise to be made between the different properties. For example, increasing the porosity in order to improve cell colonization and bioresorption, leads to a decrease in mechanical strength. Adding inorganic or organic material to improve porosity, injectability or mechanical strength can lead to a much longer setting time.

One kind of additive, surfactants, which should greatly improve some of the properties, has been quite poorly studied in this field. Surfactants are known to adsorb at liquid-liquid interfaces (typically water—oil emulsions), liquid-gas interfaces (foams) and liquid-solid interfaces (particle dispersions). Addition of a correctly selected surfactant usually stabilizes the dispersion of one phase into the other and greatly alters the texture and the rheological properties. Since calcium phosphate cements are concentrated dispersions of particles in an aqueous solution, addition of a surfactant should improve the quality of the formulation. In fact, such advantages of adding surfactants to cement dispersions are already well known in the world of concrete used for buildings. Surface agents, called air-entraining agents, bring porosity resulting in lighter cements which also show a better resistance to freeze-thaw cycles. This is related to the foaming ability of surfactants, which helps the stabilization of air bubbles in the concrete, giving, after setting, solid foams. To our knowledge, addition of surfactants to biomedical calcium phosphate cement pastes has only been described in a few studies. Planell *et al*. [9] described the addition of sodium dodecylsulfate (SDS) and hexadecyl-trimethyl-ammonium chloride (CTAC), in the proportion of 0.25% in cement powder, without pointing out any effect on porosity. Sarda, Planell, Fernandez *et al*. [10,11] again studied the addition of SDS in the liquid phase of the calcium cement up to ≈350 mM, and noted that time the formation of macropores of typically 100 μm and up to 500 μm for the highest concentrations. In the same research group, the generalization to non-ionic surfactants, such as PEO surfactants and PEO-sorbitan esters [12], or to surface-active proteins such as those of albumen [13,14], has been demonstrated. Addition of SDS was also applied by Hesaraki *et al*. to make porous cements [15]. Another way to use surfactants in cement was exploited by Bohner [16], who described the formulation of calcium phosphate emulsions, by adding both oil and a surfactant (sorbitan monooleate or a PEO surfactant). The dispersion of the oily drops in cements resulted in the formation of highly porous cements, after sintering of the hardened cement at high temperature. But this method does not rely directly on the foaming property of surfactant to create pores and is mostly used for making porous and sintered calcium phosphates.

In fact, as described above, two properties can be expected to be improved by the addition of surfactants: porosity and injectability. While an increase in porosity has been demonstrated by these former studies, an improvement in injectability was not clear, except recently with albumen proteins and sorbitan esters. However, in the first example, quite high amounts of albumen (10 wt % of the calcium phosphate powder) were required for reaching complete injection [13], and in the second example, full injectability could not be achieved [12].

In this context, our interest is focused on the addition of sugar surfactants, and more especially, sucrose surfactants, in a calcium phosphate cement, taking advantage of our good knowledge of these molecules [17]. Sucrose esters are prepared by trans-esterification of fatty acid esters with sucrose. This reaction gives surfactants which, upon hydrolysis, split in sugars and fatty acids. Today they are used as food additives (E 473) or in cosmetic and oral or topical pharmaceutical formulations. Our former experience on the stabilization of solid dispersions by these surfactants led us to expect quite good results on the texture and rheology of calcium phosphate dispersions too [18,19]. Various hydrophile-liphophile balances are available depending on the fatty acid chain length and the number of chains grafted on sucrose. Thus, we tuned this parameter and showed its effect on different properties of the cements. Compared with other surface-active agents already studied, they are non‑ionic, non‑irritating, and they are synthetic products ensuring a good reproducibility. In addition, another family of sugar surfactants, alkylpolyglucosides, has been tested. This family is more represented than sucrose esters in terms of production levels, and also displays good toxicological profiles [20].

In this part of the study, we describe the effect of these surfactants on the cement preparation (sterilization, mixing), the setting time, porosity, compressive strength and cohesion. We used a cement already available on the market (Cementek [21] and Cementek LV [22] from Teknimed) and the experiments were performed according to a quite classical approach of these systems. We show that addition of sucrose esters leads to a strong improvement of the porosity without lengthening the setting time too much. From all points of view, the quality of the formulation is better.

## 2. Results and Discussion

### 2.1. Sample preparation, mixing and sterilization

The way that samples are prepared has been shown to affect their properties, such as porosity and setting time. Thus, preliminary experiments were made in order to assess the effect of mixing and sterilization and to set up a reproducible protocol. For these preliminary tests, sucrose esters SE16P or SE11S were used. The surfactant can be added either to the liquid phase or to the calcium phosphate powder before mixing together. Addition in the liquid phase, as described by Ginebra *et al*. [12], appeared not to adapt well in the case of sucrose esters, because they are not stable in an acidic solution. Also from a practical point of view, addition to the powder appeared more judicious especially for up-scaling and long–term conditioning. Further, we observed that the foaming behavior of the paste and the amount of air bubbles introduced in the cement pastes depended on the device used for making the mixture. Making the mixture of powder and liquid in the mortar did not lead to as good a level of porosity as when it was made in a plastic bowl. We related this phenomenon to, perhaps, a stronger crushing of the paste in the mortar and some surface tension effects that could occur differently in the plastic bowl than the ceramic mortar. Given this quite significant difference for porosity enhancement between the two protocols, the one described in the experimental part was strictly followed for all samples.

We observed also that both the setting time (Table 1) and the porosity (Table 2) are affected by sterilization. The effect of sterilization on the properties of cements has been described earlier, either for the case of cements containing organic additives, which was explained by changes of the organic component induced by sterilization procedures [23], but it has also been observed quite recently by Takechi *et al*. with only mineral cements [24]. In Table 1 and Table 2, three methods have been tested: neither cement nor surfactant was sterilized (Cementek + surfactant without sterilization); cement and surfactant have been sterilized separately before mixture (Cementek + surfactant sterilized before mixture); and the mixture cement and surfactant have been sterilized after mixing (Cementek + surfactant sterilized after mixture). It can be seen that only the latter method leads to a significant decrease in setting time, with differences varying from three minutes to more than 30 minutes, compared with the first and second methods. The difference tends to increase with the amount of surfactant, since with surfactants at 2%, setting times are about 30 minutes longer if the mixture of cement and surfactant is not sterilized. Rapid control by HPLC has been made to check if sucrose esters were hydrolyzed into sucrose and fatty acid after sterilization. We did not see any difference in the chromatographic profile of the sucrose ester extracted from powder before or after sterilization. Thus the difference cannot be ascribed to a direct degradation of the surfactant. Porosity was also affected by the sterilization to a lesser extent (Table 2). Porosity tends to be slightly lower if Cementek and surfactant are sterilized together. This result is in accordance with the fact that strong degradation of the surfactant should not occur under sterilization, since the latter kept its foaming efficiency. The tendency of the cement paste to disintegrate in water before setting (see below, Table 5) is also affected by the sterilization. The paste tended to disaggregate more if cement and surfactant are not sterilized after mixing, and the tendency increases with the amount of surfactant. As a consequence, for the sake of reproducibility, the cement and surfactant mixed in a mortar have been systematically sterilized before mixing with the liquid phase.

**Table 1:** Effect of sterilization on setting times (min).

Surfactant	Cementek + surfactant without sterilization	Cementek + surfactant sterilized ***after*** mixture
none	26	18
SE11S 0.5%	23	20
SE11S 1%	27	22
SE11S 1.5%	30	25
SE11S 2%	56	29
SE16P 1%	31	24
M68EC 1%	32	24
	Cementek + surfactant without sterilization	Cementek + surfactant sterilized ***before*** mixture
SE16P 1%	31	29
M68EC 1%	32	30
	Cementek + surfactant sterilized ***before*** mixture	Cementek + surfactantsterilized ***after*** mixture
SE16P 2%	61	32
M68EC 2%	63	29

**Table 2:** Effect of sterilization on porosity (%).

Surfactant	Cementek + surfactant without sterilization	Cementek + surfactantsterilized after mixture
none	46	
SE16P 0.5%	50	50
SE16P 1%	53	51
SE16P 1.5%	55	51
SE16P 2%	57	52
SE11S 2%	58	54
M68EC 0.5%	49	48
M68EC 1%	51	50
M68EC 1.5%	52	51

### 2.2. Setting time

**Figure 1:**
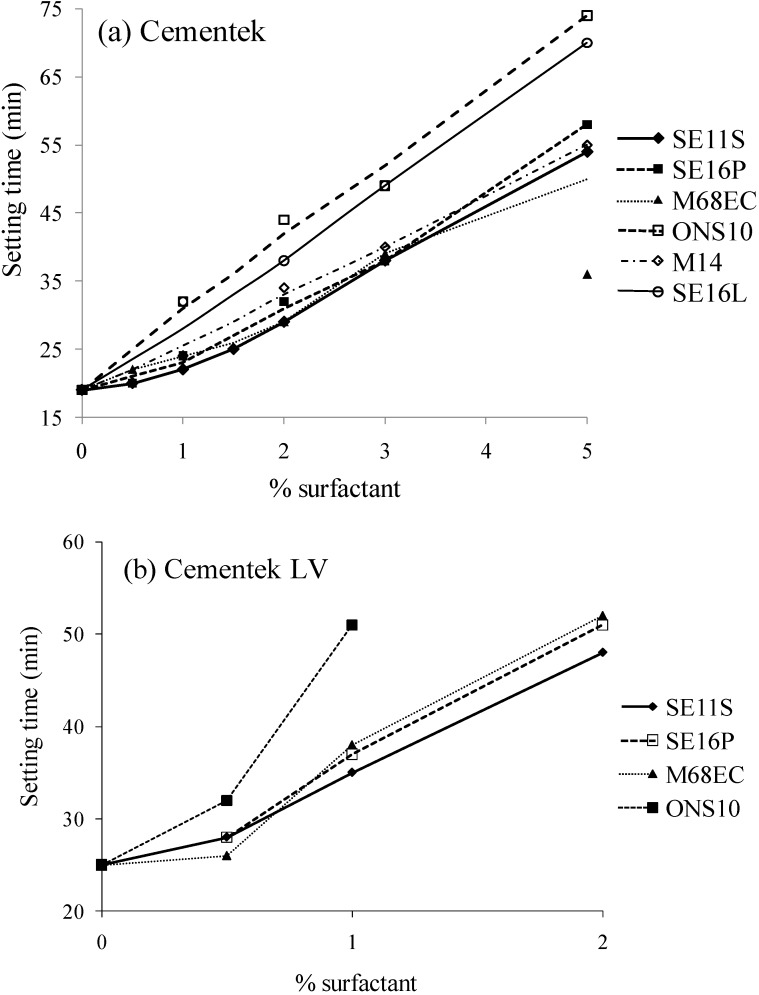
Setting time (min) (cement + surfactant sterilized after mixing). (**a**) Cementek; (**b**) Cementek LV.

The addition of various compounds, inorganic or organic, to calcium phosphate cement pastes is known to affect the setting time, sometimes to a great extent. Actually, some of them are known to inhibit the crystal growth. The setting time of the cements should be adjusted between 3 and 15 min, which corresponds to the best period of time for surgical practice. In the case of injectable cements, a longer delay can be acceptable, since it is not necessary to wait for setting before closing the wound, as long as those cements do not disintegrate *in situ* before setting. Many additives for enhancing injectability lead to long delays in setting. Additionally, by adding sugar derivatives, we expected that setting times would be delayed. Actually, the addition of table sugar (sucrose) to construction cement or concrete has long been known to significantly delay the setting [25]. Thus, setting times as a function of the nature and the amount of surfactant are reported in Figure 1a and b. Nearly all the setting times were less than 30 minutes for up to 2% of surfactant, with Cementek. Over 2% of surfactant, setting times varied from 40 minutes to more than one hour. Thus, as expected, the setting time increased with the amount of surfactant added. But it can be seen that up to 2% of surfactant can be added without lengthening the setting time too much. For Cementek LV, since the setting time of the cement without additive is longer than for Cementek, the setting times with surfactants are accordingly longer, remaining suitable with up to 1% of surfactant. Differences between surfactants can be pointed out too. Significantly longer setting times have been measured with ONS10 and SE16L, while less or similar setting times have been obtained with SE11S, SE16P, M68EC and M14. ONS10 and SE16L are both the most hydrophilic surfactants; far more than the others. It is quite interesting to note this relation between hydrophilicity of the surfactant added and a lengthening of the setting time.

### 2.3. Porosity

**Figure 2:**
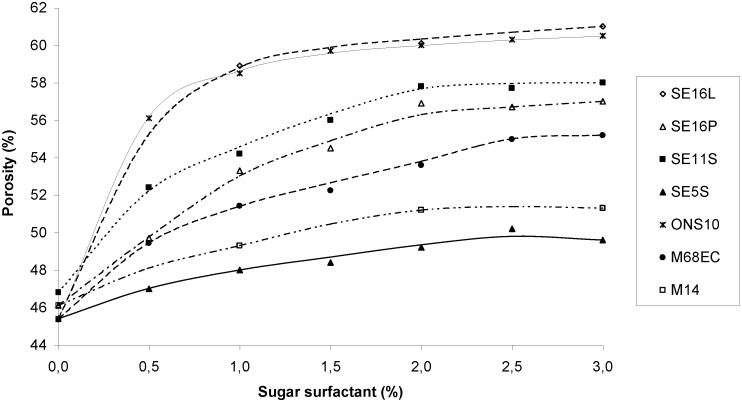
Porosity as a function of sugar surfactant measured from the volume increase of the cylinders.

From the very beginning of the work, the addition of sucrose esters showed very interesting changes in the behavior of the cement paste. After three minutes of mixing, the paste looked smoother, and was foamy and sticky. When introduced in a cylindrical mold, the paste tended to expand slightly out of the mold. The air bubbles entrapped in the cement during mixing and stabilized by the surfactant were compressed when the paste was poured in the molds. The relaxation of the bubbles pressure then led to this expansion phenomenon. At equivalent cement weight, the occupied volumes were much higher, and small holes could be seen with the naked eye all around the samples. Clearly, the addition of sucrose surfactants brought the expected air-entraining properties to the bone cements. The porosity has thus been measured as a function of the nature and the amount of additive and is reported in Figure 2. Porosity was first determined by simple measurement of the volume increase.

From these curves, the following conclusions have been drawn. First, the efficiency of the additive for inducing porosity can be classified in the order: ONS10 ≈ SE16L > SE11S > SE16P > M68EC > M14 > SE5S. Secondly, the porosity follows a quick increase of the surfactant amount at the beginning, then progresses only slightly thereafter; this effect being more pronounced for the surfactants giving the highest porosity. The effect follows the hydrophile-lipophile balance of the surfactants, since ONS10 is the more hydrophilic and SE5S the more hydrophobic, except for SE11S and SE16P that were expected in the reverse order, since SE16P is more hydrophilic than SE11S. M68EC is a mixture of about 50/50 cetylalcohol and cetylglucoside and is thus expected to be of intermediate to low hydrophilicity. The more hydrophilic the surfactant, the more foaming is expected, which conforms with the observation of porosity.

**Figure 3:**
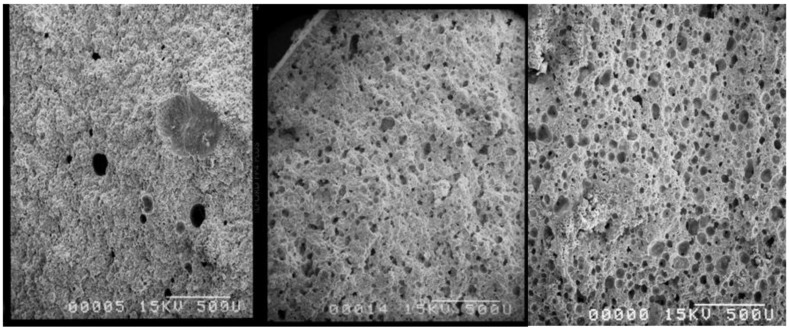
SEM of cements cross-sections: (**a**) Cementek; (**b**) Cementek + 1% SE16P; (**c**) Cementek + 2% ONS10. Bar = 500 μm.

**Figure 4:**
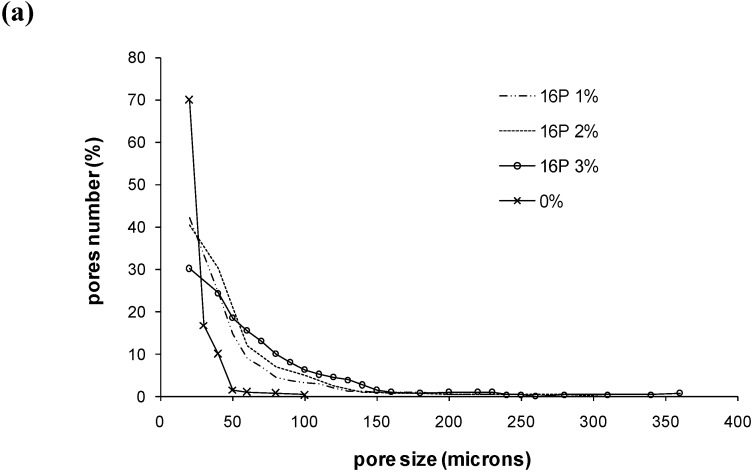

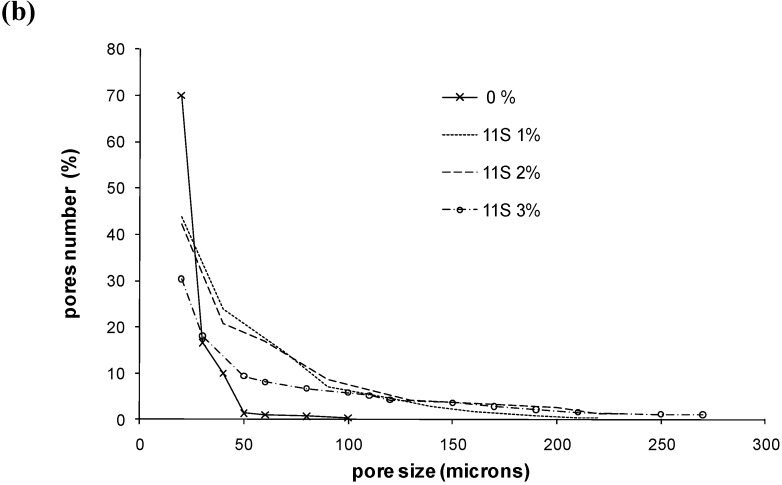
Statistical analysis of pore size by image analysis of SEM results: (**a**) SE16P; (**b**) SE11S.

Photos of cylinder cross-sections have been taken by scanning electron microscopy (SEM) and show the holes (Figure 3a–c). On the whole, pores bigger than 100 μm can be seen and many of them are interconnected, which should favor full resorption and true bone reconstruction. More precisely, image analysis of these SEM pictures allowed us to extract a statistical analysis of the pores. For SE16P and SE11S, the distributions of pore diameters have been extracted and are shown on Figure 4a and b and in Table 3 as a function of surfactant amount. Mean pore diameter, biggest pores diameters, proportion of big holes ≥100 μm, and small holes <50 μm and pore density, have been calculated. This statistical analysis showed a strong change in holes distribution with the amount and nature of surfactant. Increasing the amount of sucrose ester led to a strong decrease of the quantity of small pores and a strong increase of large pores. While in the cement without surfactant, biggest pores were about 100 μm and nearly all the pores were smaller than 50 μm, very big pores ranging from 270 to 360 μm were obtained with 3% SE11S or SE16P, and pores larger than 100 μm represented more than 25% of the pores. The biggest pores were obtained with SE16P instead of SE11S, namely, with the most hydrophilic surfactant of the two. The amount of pores, whatever their size, for a given surface increased strongly with addition of surfactant compared with the blank cement, showing a maximum at 2%, and decreasing over 2%, due to the increase of the pore size. From this analysis, pores larger than 100 μm, suitable for bone cells colonization, which are nearly absent in the cement without surfactant, represented between 10% to 30% in cements with increasing amount of SE11S or SE16P, from 1% to 3%.

**Table 3:** Statistical analysis of pore size by image analysis of SEM results.

	mean pore diameter	diameter of bigger pores	pores with diameter <50µm (%)	pores with diameter ≥100 µm (%)	Number of pores for a surface 1.8 × 1.3 mm^2^
0%	26 µm	100 µm	96.6	0.3	71
11S 1%	49 µm	220 µm	66.8	11.4	241
11S 2%	56 µm	230 µm	63.1	12.4	343
11S 3%	66 µm	270 µm	48.4	24.4	283
16P 1%	48 µm	225 µm	65.8	9.4	231
16P 2%	54 µm	280 µm	60.7	15.0	309
16P 3%	92 µm	360 µm	27.9	33.1	206

### 2.4. Compressive strength

**Figure 5:**
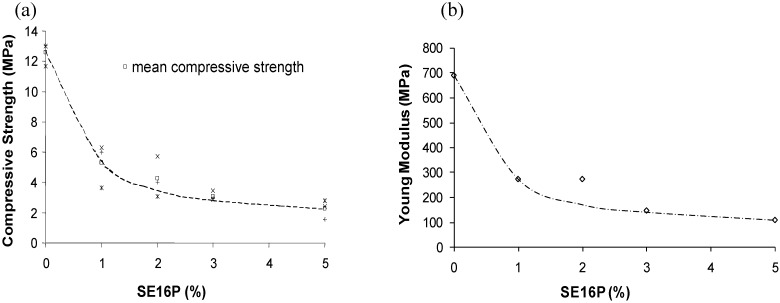
(**a**) Compressive Strength and (**b**) Young’s Modulus for compression as a function of SE16P concentration.

As a consequence of porosity increase, changes in compressive strength have been measured for samples with SE16P as surfactant. As expected, compressive strength decreased quite strongly with the formation of pores, from nearly 12 MPa to 4 MPa (Figure 5a). This sharp decrease occurred from 1% surfactant, then did not decrease again so sharply when increasing the surfactant up to 5%. The Young’s modulus followed the same pattern (Figure 5b).

### 2.5. Cohesion of the pastes

Since calcium phosphate cement is designed to come in contact with body fluids before setting, the cohesion of the paste must be strong to avoid swelling of implants or dispersion of cement particles in any place, which can cause unexpected biological response *in vivo*, including severe inflammation or thromboses [26,27,28,29,30,5]. In order to assess the effect of our additives on cohesion, the paste was poured into water five minutes and 30 seconds after the beginning of mixing and disintegration was observed. The results have been expressed qualitatively: +++ was assigned to samples which showed a very quick and complete disintegration, and --- to samples showing no release of visible particles in water after two hours. These results are reported in Table 4 (Cementek, ball shaped) and Table 5 (Cementek LV, injection in water). The surfactants can be ranked in the following order from best cohesion to complete disintegration: SE16P > M68EC ≈ SE11S > M14 >> SE16L >> ONS10. While the two hydrophilic surfactants, ONS10 and SE16L, showed very good results for increasing porosity, the resulting pastes displayed very poor resistance to disintegration in water. For both these surfactants, the paste very quickly disaggregated in water by releasing air bubbles. Whereas SE16P allowed a good increase of the porosity, the paste also kept a good texture and resistance to disintegration in water, even at high level of surfactant. Clearly a compromise has to be found between porosity enhancement and cohesion. We should discard the use of SE16L and ONS10 and we should keep SE16P as a good compromise. Otherwise, cohesion is not only inversely related to porosity. It can be related to adhesive properties of sucrose esters pastes, which could explain why SE16P kept a good cohesion even with the increasing number of pores.

**Table 4:** Disintegration of Cementek in water (“ball” shaped).

Cementek	SE11S	SE16P	SE16L	M14	M68EC	ONS10
**0%**	**---**					
**1%**	**---**	**---**	**+**	**---**	**---**	**+++**
**2%**	**---**	**---**	**+**	**--**	**---**	**+++**
**3%**	**--**	**---**	**++**	**+**	**---**	**+++**
**5%**	**+**	**---**	**++**	**--**	**---**	n.d.
**10%**	n.d.	**-**	n.d.	n.d.	n.d.	n.d.
**20%**	n.d.	**+** (swelling)	n.d.	n.d.	n.d.	n.d.

**Table 5:** Disintegration of Cementek LV in water, after injection through a syringe (“worm” shaped). With cement and surfactant sterilized together: “S”, or not sterilized together: “NS”.

Cementek LV		SE11S	SE16P	M68EC	ONS10
**0%**		**---**			
**0.5%**	**S**	**---**	**---**	**--**	**+++**
	**NS**	**--**	**-**	**-**	**+++**
**1%**	**S**	**---**	**---**	**--**	n.d.
	**NS**	**-**	**-**	**+**	n.d.
**1.5%**	**S**	**---**	**---**	**-**	n.d.
	**NS**	**-**	**-**	**+**	n.d.
**2%**	**S**	**--**	**---**	**+**	n.d.
	**NS**	**+**	**+**	**+++**	n.d.

## 3. Experimental Section

*Materials*: Calcium phosphate cements have been prepared from the ready-to-use kit Cementek and Cementek LV (Teknimed, Toulouse, France). These preparations include a calcium phosphate powder (tricalcium phosphate, tetracalcium phosphate and sodium glycerophosphate) and an aqueous solution (phosphoric acid and calcium hydroxide). Food grade sucrose esters (sucrose laurate -SE16L-, sucrose palmitate -SE16P-, sucrose stearate -SE11S, medium subsitution degree-, sucrose stearate -SE5S, high substitution degree, hydrophobic-) have been kindly supplied by Stéarinerie Dubois (France). Alkylpolyglucosides (Montanov 68EC—M68EC, mixture of cetylalcohol and cetylglucoside, Montanov 14—M14, mixture of myristylalcohol and myristylglucoside-, Oramix NS10—ONS10, decylglucoside-) have been kindly supplied by Seppic (France).

*Sample preparation*: The desired amount of sugar surfactant was added to the calcium phosphate powder, and the powders crushed together in a mortar for two minutes. The powder mixture was transferred in the plastic bowl provided in the kit, and the aqueous calcium phosphate solution was added, according to the proportions given by the manufacturer. More precisely, samples were made from 2.00 g of powder and 0.86 g of solution. The proportion of sugar surfactant added was defined as the weight percent of the whole mixture powder + liquid, and the mass of surfactant was neglected in the calculation. Typically, cement with 1% sugar surfactant is composed of 2.00 g powder, 0.86 g solution and 29 mg surfactant. After addition of the liquid, the mixture was mixed for three minutes with a small flat spatula during which a smooth paste is formed. After this delay, the paste is used for various experiments. For all experiments, the time t = 0 corresponds to the mixing of cement with the acidic solution. The powders were sterilized by gamma irradiation, before or after mixing with additives, according to the conditions described in the discussion, and the liquid phase was also sterilized by gamma irradiation before use.

*Setting time*: The setting time was measured by indentation according to an adaptation of the Gillmore needle method. The paste was poured into a plastic mold of 8 mm diameter and 8 mm depth. A needle with a flat tip (surface = 0.785 mm^2^) was driven over 5 mm into the setting cement, every minute. The resulting force was monitored with a TAXT2 Texture Analyzer from Stable Microsystems. The cement is considered to be set when the pressure reaches 0.6 kg/mm^2^ (5.9 MPa).

*Porosity*: Porosity was quantified by two methods. In the first method, calibrated cylindrical samples were prepared by pouring the paste into cylindrical molds of 9.0 mm diameter. After two hours of setting, cylinders were removed from the molds and incubated at 37 °C in pure water for seven days to ensure transformation in hydroxyapatite (it was checked by X-Ray on a few samples that the transformation in HAP was only partial in the presence of sucrose esters). The samples were washed with acetone and air–dried for seven days. The mass (m, in g), the volume (V, cm^3^) were measured and density (d, g/cm^3^) calculated and compared with the density of pure hydroxyapatite, which is 3.15 g/cm^3^. The porosity percent corresponding to the ratio {volume of pores/total volume} × 100 was thus calculated as: p = (1−d/3.15) × 100.

In the second method, sections of the same cylinders were observed by scanning electron microscopy (SEM) on a Hitachi S450 and after metallization Au-Pd. Image Analysis using the software Image J was made on the photos. After preparation of the photos (selection of a surface of reference, contrast enhancement, manual cut of fused holes), the number of holes, surfaces, and perimeters were extracted. From these data, a statistical analysis giving the number of holes as a function of the mean diameter (perimeter/π) was plotted.

*Mechanical tests*: Compressive strength was measured on cement cylinders prepared as described in the section “Porosity”, but with home-made plastic molds (9 mm internal diameter, 10 mm height) made of three parts—two hemi-cylinders and a ring—enabling easy removal and a precise dimension of the cylinders. The cylinder dimensions were measured and varied from 8.68 to 9.10 mm for the diameter, and from 8.6 to 11.7 mm for the length. Three samples were tested for each concentration of additive. A force was applied on the cylinder until crushed, using a mechanical testing machine (Hounsfield). The curves were reported as stress (Force/Surface of cylinder, MPa) as a function of displacement (mm). Young’s moduli were measured as the slope of the curve multiplied by the cylinder length in the linear region.

*Cohesion*: Cohesion of the paste when poured in water was assessed visually. With Cementek, a ball of cement was prepared from the paste (with 2.00 g of powder), and at t = 5’30”, the ball was poured in pure water at room temperature. After two hours, the aspect of the sphere was qualitatively classified ---/--/-/+/++/+++ indicating increasing disintegration. The same kind of experiments has been made on some samples extruded from a syringe (with the tip, but no needle) in water, with Cementek LV. The cohesion of the resulting “worms” was reported.

## 4. Conclusions

In this study, we showed how the addition of sugar surfactants and, more especially, sucrose fatty acid esters improved several properties of calcium phosphate cements. They allowed the formation of pores larger than 100 microns by a mechanism of air entrainment and air bubble stabilization, without excessive lengthening of the setting times. Good texture and cohesion have also been obtained for the cement pastes before setting. Compared with other methods used for enhancing porosity, the addition of surfactants appeared very easy to set up, with less risk of gas release, and no impairment of the setting time. A simple mixing of the solids―cement powder and surfactant powder―is efficient for providing the desired properties and compatible with long-term storage.

## References

[1] Dorozhkin S.V. (2009). Calcium orthophosphates in nature, biology and medicine. Materials.

[2] Dorozhkin S.V. (2008). Calcium orthophosphate cements for biomedical application. J. Mater. Sci..

[3] Giannoudis P.V., Dinopoulos H., Tsiridis E. (2005). Bone substitutes: An update. Injury, Int. J. Care Injured.

[4] LeGeros R.Z. (2008). Calcium phosphate-based osteoinductive materials. Chem. Rev..

[5] Bohner M., Gbureck U., Barralet J.E. (2005). Technological issues for the development of more efficient calcium phosphate bone cements: A critical assessment. Biomaterials.

[6] Lewis G. (2006). Injectable bone cements for use in vertebroplasty and kyphoplasty: state-of-the-art review. J. Biomed. Mater. Res.-Part B.

[7] Habib M., Baroud G., Gitzhofer F., Bohner M. (2008). Mechanisms underlying the limited injectability of hydraulic calcium phosphate paste. Acta Biomater..

[8] Biresaw G., Rudnick L.R. (2009). Surfactants in lubrication. Lubricant Additives: Chemistry and Applications.

[9] Ginebra M.P., Boltong M.G., Fernandez E., Planell J.A., Driessens F.C.M. (1995). Effect of various additives and temperature on some properties of an apatitic calcium phosphate cement. J. Mater. Sci.: Mater. Med..

[10] Sarda S., Fernandez E., Nilsson M., Planell J.A. (2002). Influence of air-entraining agent on bone cement macroporosity. Key Eng. Mater..

[11] Sarda S., Nilsson M., Balcells M., Fernandez E. (2003). Influence of surfactant molecules as air-entraining agent for bone cement macroporosity. J. Biomed. Mater. Res.-Part A.

[12] Ginebra-Molins M.P., Planell-Estany J.A., Gil-Mur F.X. (2006). Injectable, self-setting calcium phosphate foam. Patent.

[13] Ginebra M.P., Delgado J.A., Harr I., Almirall A., Del Valle S., Planell J.A. (2007). Factors affecting the structure and properties of an injectable self-setting calcium phosphate foam. J. Biomed. Mater. Res. Part A.

[14] Delgado J.A., Harr I., Almirall A., del Valle S., Planell J.A., Ginebra M.P. (2005). Injectability of a macroporous calcium phosphate cement. Key Eng. Mater..

[15] Hesaraki S., Nemati R. (2009). Cephalexin-loaded injectable macroporous calcium phosphate bone cement. J. Biomed. Mater. Res.-Part B.

[16] Bohner M. (2001). Calcium phosphate emulsions: possible applications. Key Eng. Mater..

[17] Muller A.S., Gagnaire J., Queneau Y., Karaoglanian M., Maître J.P., Bouchu A. (2002). Winsor behaviour of sucrose fatty acid esters: Choice of the cosurfactant and effect of the surfactant composition. Colloids Surf. A.

[18] Fitremann J., Queneau Y., Maître J.P., Bouchu A. (2007). Co-melting of solid sucrose and multivalent cations soaps for solvent-free synthesis of sucrose esters. Tetrahedron Lett..

[19] Datsyuk V., Landois P., Fitremann J., Peigney A., Galibert A.M., Soula B., Flahaut E. (2009). Double-walled carbon nanotubes dispersion via surfactant substitution. J. Mater. Chem..

[20] Von Rybinski W., Hill K. (1998). Alkyl polyglycosides: properties and applications of a new class of surfactants. Angew. Chem. Int. Ed..

[21] Lacout J.L., Hatim Z., Frèche-Botton M. (1998). Procédé de préparation d'un biomatériau à base d'hydroxyapatite, biomatériau obtenu et application chirurgicale ou dentaire. Patent.

[22] Lacout J.L., Frèche M., Gonçalves S., Rodriguez F. (2000). Procédé de préparation d'un matériau pâteux phosphocalcique injectable etc.. Patent.

[23] Zahraoui C., Sharrock P. (1999). Influence of sterilization on injectable bone biomaterials. Bone.

[24] Takechi M., Miyamoto Y., Momota Y., Yuasa T., Tatehara S., Nagayama M., Ishikawa K. (2004). Effects of various sterilization methods on the setting and mechanical properties of apatite cement. J. Biomed. Mater. Res. Part B.

[25] Garci Juenger M.C., Jennings H.M. (2002). New insights into the effects of sugar on the hydration and micostructure of cement pastes. Cem. Concr. Res..

[26] Miyamoto Y., Ishikawa K., Takechi M., Toh T., Yuasa T., Nagayama M., Suzuki K. (1999). Histological and compositional evaluations of three types of calcium phosphate cements when implanted in subcutaneous tissue immediately after mixing. J. Biomed. Mater. Res. Part B.

[27] Bohner M., Doebelin N., Baroud G. (2006). Theoretical and experimental approach to test the cohesion of calcium phosphate pastes. Eur. Cell. Mater..

[28] Khairoun I., Driessens F.C.M., Boltong M.G., Planell J.A., Wenz R. (1999). Addition of cohesion promotors to calcium phosphate cements. Biomaterials.

[29] Pioletti D.P., Takei H., Lin T., Van Landuyt P., Ma Q.J., Kwon S.Y., Paul Sung K.L. (2000). The effects of calcium phosphate cement particles on osteoblast functions. Biomaterials.

[30] Gauthier O., Bouler J.M., Weiss P., Bosco J., Aguado E., Daculsi G. (1999). Short-term effects of mineral particle sizes on cellular degradation activity after implantation of injectable calcium phosphate biomaterials and the consequences for bone substitution. Bone.

